# Supplementary material for: QTL Mapping and Transcriptome Analysis Reveal Candidate Genes Regulating Seed Color in Brassica napus
Source: Int J Mol Sci. 2023 May 25;24(11):9262. doi: 10.3390/ijms24119262 (PMC10253132; doi:10.3390/ijms24119262)
Supplement: Supplementary file 1 [file ijms-24-09262-s001.zip › FigureS.pdf]

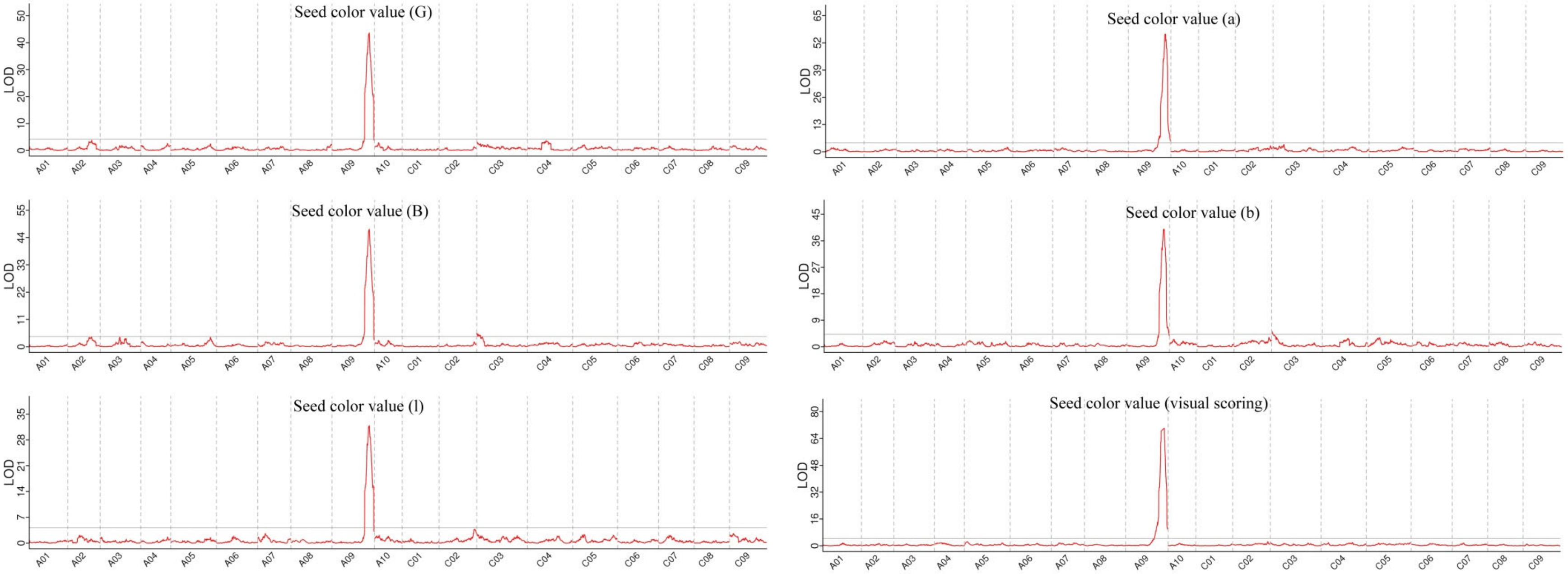

Figure S1. LOD distributions on chromosomes according to different seed color values.

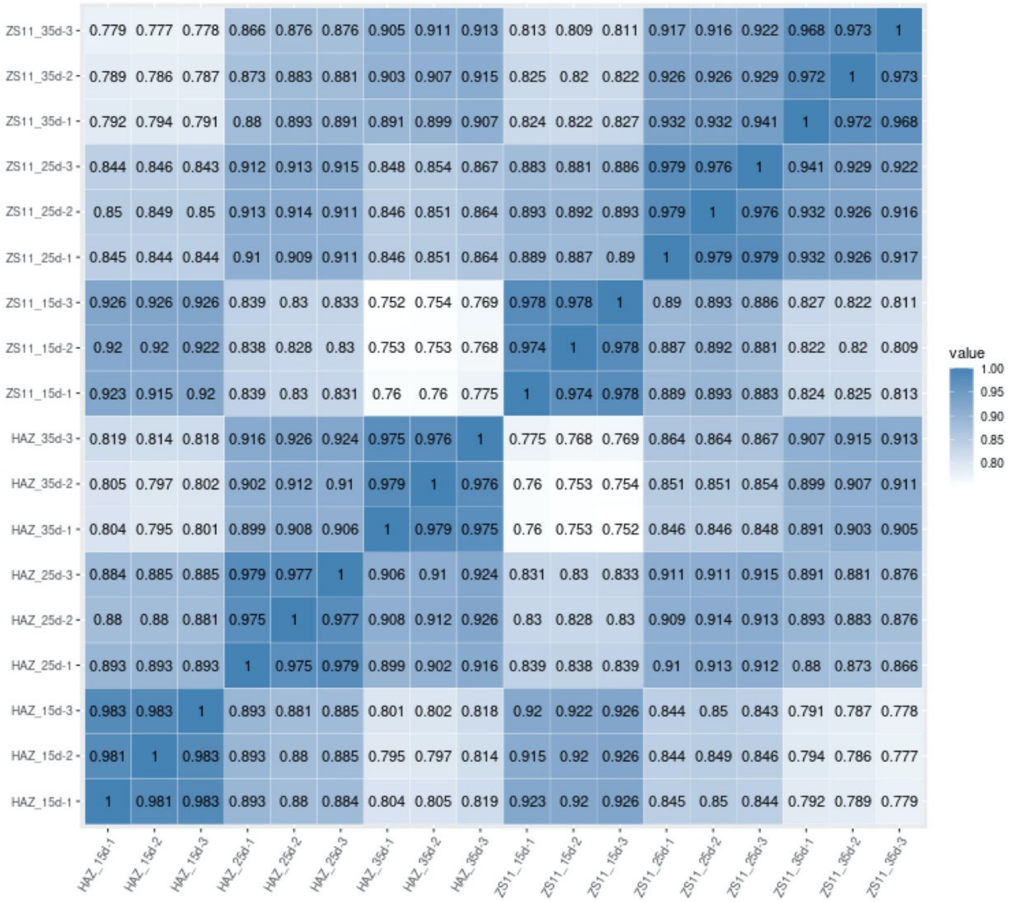

Figure S2. Pearson correlation coefficients between samples.

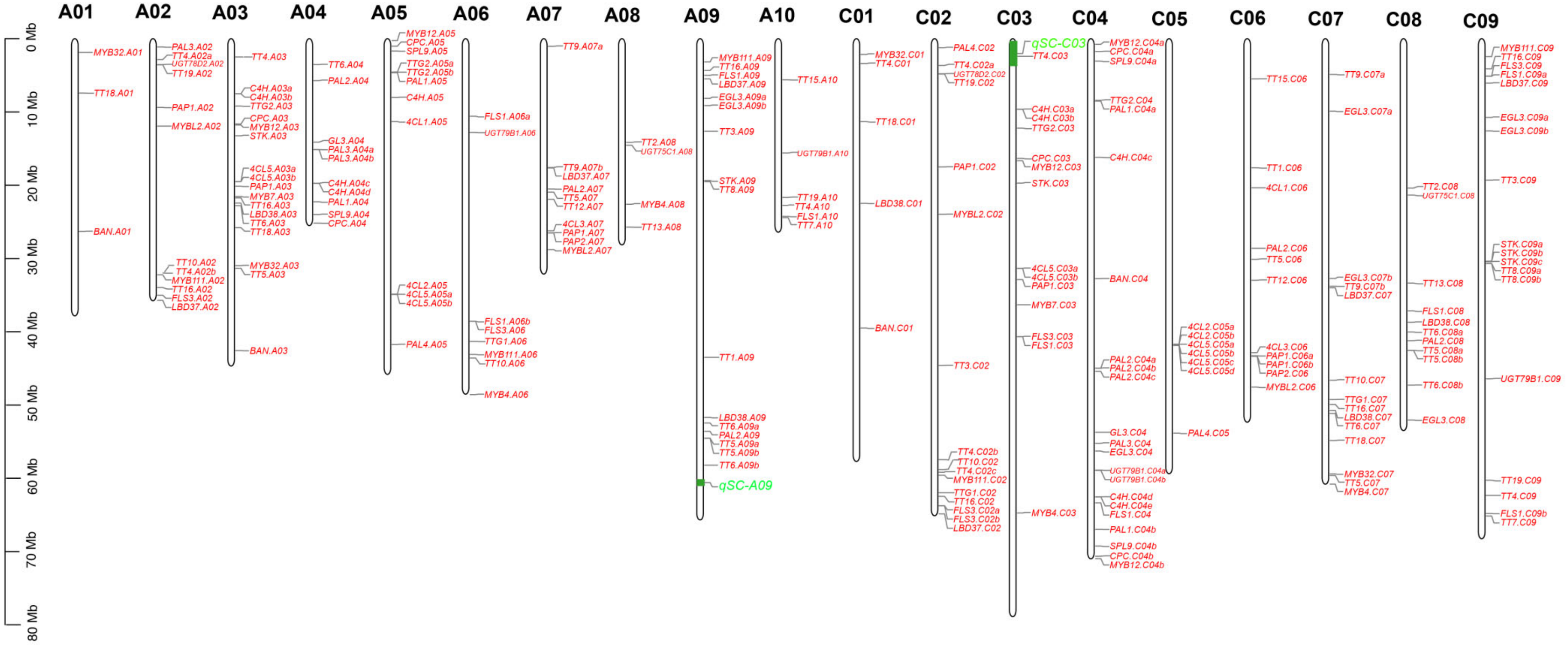

Figure S3. Distribution of flavonoid-related genes and QTL intervals for seed color on chromosomes.

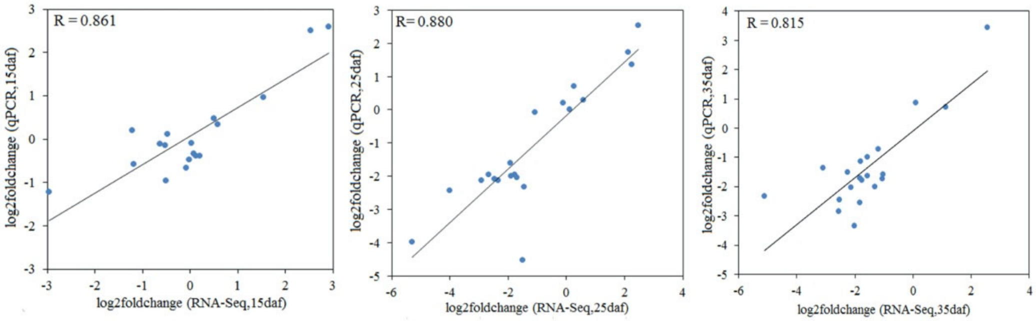

Figure S4. Correlations of gene expression changes from qPCR and RNA-seq methods at each seed developmental stages (15, 25, 35, 35 daf).
